# Current Milestones Assessment Practices, Needs, and Challenges of Program Directors: A Collective Case Study in a Pediatric Hospital Setting

**DOI:** 10.7759/cureus.14585

**Published:** 2021-04-20

**Authors:** Kadriye O Lewis, Susan B Hathaway, Denise Bratcher, Douglas Blowey, Jane F Knapp

**Affiliations:** 1 Department of Pediatrics, Graduate Medical Education, Children’s Mercy Hospital, University of Missouri Kansas City School of Medicine, Kansas City, USA; 2 Nephrology, Department of Pediatrics, Children’s Mercy Hospital, University of Missouri Kansas City School of Medicine, Kansas City, USA

**Keywords:** pediatric milestones, milestones assessment and evaluation, milestones challenges, program needs, program directors’ assessment practices

## Abstract

Introduction

Accreditation Council for Graduate Medical Education's (ACGME's) Milestones assessment requirement has placed new demands on Program Directors (PDs), especially those with limited knowledge of assessment and evaluation activities. There is a lack of clarity on how Program Director (PDs)/Associate PDs (APDs) are effectively implementing milestones assessment and evaluation practices in the Graduate Medical Education programs. The purpose of this study was to investigate current assessment practices, needs, and challenges of PDs in implementing milestones assessment within their residency and fellowship programs in a pediatric hospital setting.

Methods

This study used a collective case study approach to obtain information from PDs, APDs, and Clinical Competency Committee (CCC) Chairs in 19 graduate programs at a pediatric hospital. We used structured meetings with planned agendas and a pre-formatted template to itemize program needs/difficulties/challenges in the milestone assessment. We used cross-case thematic content anal­ysis to identify categories and themes to compare differences and commonalities across programs.

Results

A total of 38 PDs, APDs, and CCC Chairs from 19 different specialties/subspe­cialties participated in this study. Thirteen types of assessment and evaluation tools were consistently used across programs. Three categories emerged in relation to those assessment and evaluation types (direct, indirect, and multi-source). Rotation evaluation (84.2%), direct observation (73.2%), and 360-degree assessment (68.4%) were primarily used for measuring patient care among the six core competencies. Programs’ needs varied from curriculum and assessment tool development to alignment of milestones items, and to creating a sys­tematic assessment management plan. The most common challenges were difficulties related to logistics and tracking of evaluation in the survey management system (52.6%), challenges with time management (47.3%), and difficulty in determining and interpret­ing the milestones’ numbers and levels (31.5%).

Conclusions

Milestones assessment and evaluation in medical education can be a challenge, but a priority for many training programs. Our study indicated that milestones assessment and evaluation in medical education are far more com­plex than we expect. Multiple assessment methods must be utilized to evaluate all essential competencies for accurate measurement of trainees’ performance abilities. Our study uncovered several issues PDs faced during the implementation of milestones assessment and needs and challenges.

## Introduction

In 1999, the Accreditation Council for Graduate Medical Education (ACGME) introduced general competencies for all residents in six areas that included patient care, medical knowledge, professionalism, interpersonal and communication skills, systems-based practice, and practice-based learning and improvement [1]. After implementation of the six core competencies that became mandatory for residency programs in 2002, the ACGME shifted to assessing residents and residency program performance based on competency-based objectives and outcome data. To support the goal of competency-based assessments, Milestones development began in 2009 as a partnership between ACGME and the American Board of Medical Specialties [2]. The development phase used literature reviews, theories and models, and narrative anchor assessment approaches to create milestones for each of the ACGME sub-competencies for pediatrics [3,4]. However, this project created challenges for some training programs that needed more complex forms of assessment (e.g., unsupervised practices and/or procedural tasks that require multi-level assessment in various setting or skill-based assessment that requires measuring multiple integrated abilities such as knowledge, critical thinking, problem-solving, and decision-making skills in a given domain) and lacked proper measures reflecting the six competencies in a reliable or valid way [1]. While some of the competencies were easier to assess through valid and reliable testing (e.g., National Board Scores, Written Examinations, In-Training Examination Scores), others would be more challenging to assess as in the study conducted by Natesan et al. [5] reporting that the competencies of professionalism, interpersonal, and communication skills can be hardly differentiated in the mind of evaluators. Furthermore, some other competencies were more subjective in nature (e.g., systems-based practice and practice-based learning and improvement) and posed additional problems from an assessment perspective (e.g., competencies that are hard to observe and infrequent opportunities to assess and/or milestones that are not covered in a specific curriculum) [6].

Milestones, derived from the Dreyfus model of expertise using the five-level framework (Novice, Competent, Proficient, Expert, and Master) [7], were integrated across all accredited specialties on July 1, 2014. However, the Milestone framework and its implementation placed new demands on Program Directors (PDs) to develop assessment approaches that better align with Milestones. Some of the challenges reported from PDs in a pre-workshop survey and during two workshops presented by the authors at national meetings include: (i) PDs and medical faculty struggle with time management in applying a fair and meaningful assessment in a busy clinical setting; (ii) most of the assessment tools (i.e., evaluation forms and surveys) lack validity and reliability, largely because they are poorly developed and subjective, and fail to assess the skills and behaviors defined in the Milestones; (iii) faculty evaluators are unwilling or unable to devote sufficient time for resident assessments; (iv) evaluations and surveys, in general, do not always provide useful or meaningful data; (v) not surprisingly, those challenges created difficulties in successfully implementing the Milestones assessment.

Prior literature has focused primarily on describing the Milestone framework and performance-based assessment, including possible measurement tools and tool development or application of Milestones [2,3,8]. To the best of our knowledge, no studies have specifically investigated the assessment and evaluation practices of a large group of diverse PDs. There is also a lack of clarity on how PDs are effectively implementing Milestone assessment strategies. Moreover, there is no uniformity in the Milestone assessment procedures regarding different specialties and sub-specialties.

In brief, challenges and difficulties are present in the current Milestones implementation by Graduate Medical Education (GME) programs as well as a lack of formal training in assessment and evaluation methodologies of PDs and faculty. Therefore, we aimed to create a comprehensive, uniform, and centralized system that could focus our educational activities and reduce redundancies and excesses in new tool developments, expenditures, and workload related to the assessment and evaluation system. The purpose of this study was to investigate current assessment practices as well as identify the needs and challenges of the PDs in implementing Milestones assessment in a pediatric hospital setting.

## Materials and methods

This study used a collective case study approach to obtain information from PDs, associate PDs (APDs), and Clinical Competency Committee (CCC) Chairs regarding the Milestone assessment practices in 19 graduate programs at Children’s Mercy Hospital (CMH) in Kansas City, Missouri. We selected this approach because we wanted to examine each program’s findings within their context separately before making comparisons across programs. All 19 programs under this study had been using multiple tools for various types of assessment and evaluation methods to measure residents’ and fellows’ performance as part of the Milestones assessment requirements as defined by the ACGME (Appendices). We use the New Innovations™ survey platform (New Innovations, Inc., Uniontown, OH) for our Milestones assessment and evaluation activities that include customizable Milestone reports. All forms are loaded into this web-based software application to monitor and manage residents’ and fellows’ performance evaluations.

We obtained CMH Institutional Review Board approval prior to the study, including written consent from the participants during data collection.

Data collection

We designed a pre-formatted template as a focused conversation guide for the data collection. This template contained six questions to itemize PDs’ current assessment practices, program needs, and difficulties or challenges regarding the Milestone assessment (Appendices). A feasibility pilot of this template with one program resulted in a smooth process. To initiate our conversation with each program, we set up a private meeting in a reserved room inviting PDs, APDs, and CCC Chairs from 19 graduate programs. The second co-author (SBH) typed computer notes into the template for the conversation with PDs/APDs/CCC Chairs in real-time. We did not audio record any meetings to create a more comfortable atmosphere and eliminate participants’ anxiety that could be produced by recording devices. The total meeting time with each program was between one and two hours. Written notes were sent to each program to ensure the accuracy of the information. We completed data collection between 2015 and 2016.

Data analyses

The written text data from the meetings were coded by two coders trained in the coding scheme. We used thematic content analysis to identify categories and themes [9-13]. Themes were identified both inductively and deductively by strongly linking to the data themselves [10]. To eliminate biases, dominant themes were identified in the data through open coding [13]. This identification linked and reorganized themes to develop a dominant structure that evolved into a conceptual framework. An additional facilitator consolidated the data coding process until all codes were agreed upon. In addition, a cross-case thematic structure was created to check categories and themes for each program as well as to compare differences and commonalities across programs. Charts and tabulations were used to further categorize and quantify the data. This process aimed to increase contextual validity [13,14].

## Results

A total of 38 PDs, APDs, and CCC Chairs from 19 specialties/sub-specialties from the GME Programs participated in this study (for analysis and discussion, PDs, APDs, and CCC Chairs were combined.) All programs were using Milestone assessment to measure the six core competencies as required by ACGME. While the majority of the programs were using the New Innovations™ survey platform, a few programs reported that they were using hard copy forms to gain feedback from patients and families.

Current assessment practices

Our study found that all 19 programs had been using multiple methods to teach and evaluate the six core competencies of ACGME: (i) patient care (PC), (ii) medical knowledge (MK), (iii) practice-based learning and improvement (PBLI), (iv) systems-based practice (SBP), (v) interpersonal and communication skills (ICS), and (vi) professionalism (PROF). A total of 13 types of assessment and evaluation methods were consistently used across 19 programs (Table 1). The following three categories emerged through the thematic content analysis of those methods: (1) *Direct Assessment*: Direct measures were used to provide evidence of medical trainees’ professionally judged performances in real-time by: (i) directly observed achievement of expected outcomes, and (ii) demonstrations of abilities in the context of knowledge and skills gained during rotations and/or throughout the program completion. Performance on internal or external written examinations or licensing (board reviews) were also considered as direct assessment indicators against core competencies of ACGME. (2) *Indirect Assessment*: In indirect measures, faculty inferred actual medical trainees’ abilities, knowledge, and values rather than observed direct evidence of learning or achievement. Surveys, rotations, and conference evaluations were categorized as indirect assessments since they were used to obtain perceived learning experiences of medical trainees and their impressions or opinions about rotations, programs, and learning outcomes. (3) *Multi-Source Assessment*: This type of assessment involves self-assessment and assessments by others (peers, team members, and faculty members) to gain feedback on an individual’s behaviors or performance in a particular environment. Each type of assessment and evaluation method was described with the usage percentage as shown in Table 1.

**Table 1:** Types of assessment and evaluations used by the graduate programs. PC: patient care, MK: medical knowledge, PBLI: practice-based learning and improvement, ICS: interpersonal and communication skill, PROF: professionalism.

Types of assessment and evaluations	Description	Highest usage for a specific competency n: 19 n (%)	Average use across six competencies combined %
Direct evaluation			
Direct observation	To assess medical trainees in their professional settings (inpatient and clinic) regarding their physical examination, history taking, medical decision making, time management, professionalism, and interpersonal communication skills.	14 (73.7) (PC)	49
Discrete activity assessment	To evaluate the continuous or episodic assessment of learning through ongoing and enforcement task-related discrete activities (specific training opportunities such as boot camp simulation assessment, simulation lab assessment of medical decision making using structured scenarios, ACGME global procedural checklist, Surgical Council on Resident Education completion).	10 (52.6) (PC)	18
In-training/ written examination	To assess each trainee’s progress from year to year, as well as to obtain data to compare performance with national peer groups (included all subspecialty In-Training Examination (SITE), Residency In-Service Training Examination (RITE), General Pediatrics In-Training Examination (ITE), Clinical Research Course written final examination, and Mock board reviews).	11 (57.9) (MK)	11
Scholarly product review	To assess trainees’ creative, peer-reviewed, and publicly disseminated achievements through national or international conferences (research and /or the Quality Improvement program resulting in publication in an academic journal, a trainee’s achievement of an advanced degree, and QI mentor evaluation).	4 (21.1) (PBLI)	10
Indirect evaluation			
Rotation evaluation	A summative assessment of the trainee’s performance in meeting the learning objectives of a specific rotation or experience. This could include a combination of other assessments.	16 (84.2) (PC)	61
Conference evaluation	To assess the trainees' e-content and delivery of teaching activities (noon/case conferences, journal club meetings, Professor Rounds, and Morbidity and Mortality conferences).	12 (63.2) (PBLI)	25
Clinical review	To evaluate the clinical data review to document scientific and regulatory dialogue as well as share a review with the clinical team and other clinical key staff to develop complete and scientifically valid review perspectives (recordings from Contact Center, faculty reviews and edits outpatient and/or inpatient notes, and surgical procedures case logged on ACGME website).	11 (57.9) (PC)	19
Multi-source evaluation			
360-degree assessment	To obtain feedback from the multidisciplinary team on the professional growth, interpersonal skills, and performance of the trainees as clinicians, colleagues, and peers. The evaluators of 360 assessments have included clinic and floor nurses, staff, social workers, orthoptists, therapists, psychologists, prosthetists, nutritionists, speech pathologists, and technical staff.	17 (89.5) (PROF)	58
Non-rotation faculty assessment	To obtain indirect feedback about trainees in the form of oral and written communications to the PDs/APDs from both inside and outside of the hospital or external agencies (anecdotal information from referring physicians, discussion after tumor board, patient advocate complaint, community provider, and consensus evaluation).	11 (57.9) (PROF)	54
Informal feedback	To obtain informal, real-time feedback from various sources (PDs, APDs, CCC) to review the progress of trainees.	12 (63.2) (PC)	41
Patient feedback	To solicit views/feedback, and opinions of patients, families, and caregivers regarding community providers’ communication skills and professionalism, including appointment, scheduling, and wait time.	7 (36.8) (PROF, ICS)	18
Peer assessment	To obtain professional competencies to provide formative feedback by residents and/or fellows.	7 (36.8) (ICS)	18
Self-assessment	To capture information on how trainees feel about progressing in the program as well as identifying areas for improvement regarding their Individualized Learning Plans (ILP). They were also expected to complete a biannual structured review of self-assessment exercise with PDs regarding their goal setting for the next 6 months and an evaluation of how they had worked on their goals the past 6 months.	9 (47.4) (PBLI)	17

In Table 1, to obtain the results of the highest and average usage of the specific assessment and evaluation methods for six competencies across 19 programs, we first calculated the percentage for each competency for each type of assessment and evaluation method for each program. We then combined all six core competencies for each type of method to calculate the average use of the specific tool/method.

The results showed that PBLI was measured using a Conference Evaluation tool in 12 programs (63.2%). MK was measured using Written Examinations and In-Training Examination Scores in 11 programs (57.9%). PC was measured with a Direct Observation method in 14 programs (73.7%). Among those 13 types of assessment and evaluation methods, the top five most frequently used to measure medical trainees’ performance were (sorted from highest to lowest percentage): (i) rotation evaluation 61%, (ii) 360-degree evaluation 58%, (iii) non-rotation faculty assessment 54%, (iv) direct observation 49%, and (v) informal feedback, 41%.

We also broke down the analysis results of evaluation methods into each competency across all 19 programs to determine the frequency of evaluation methods. Among the top five evaluation methods, 360-degree evaluation was the most frequently used tool to measure the competency areas of PROF, ICS, and PC (Table 2).

**Table 2:** Assessment and evaluation methods used in high frequency (19 programs). PC: patient care, PBLI: practice-based learning and improvement, ICS: interpersonal and communication skill, PROF: professionalism, SBP: systems-based practice.

Order	Evaluation methods	Competency areas	Frequency n: 19 n (%)
1	360-degree evaluation	PROF	17 (89.5)
2	360-degree evaluation	ICS	16 (84.2)
2	Rotation evaluation	PC	16 (84.2)
3	Direct observation	PC	14 (73.7)
4	360-degree evaluation	PC	13 (68.4)
4	Rotation evaluation	PROF	13 (68.4)
5	Direct observation	SBP	12 (63.2)
5	Informal feedback	PC	12 (63.2)
5	Conference assessment	PBLI	12 (63.2)
5	Rotation evaluation	SBP	12 (63.2)

In addition, across all 19 programs, an average of five to six evaluation types/methods were used to measure PC competency, while comparatively fewer methods (three to four on average) were used to measure either MK or SBP. Also, the majority of the programs were using the New Innovations platform while a few programs reported that they were using hard copy forms to gain feedback from patients and families.

Needs to be Specified by Programs

The data analysis showed that similar assessment and evaluation tools were consistently needed across all programs. Alignment between Milestones descriptions and competency measurement methods/items was the primary need (repeated frequently) when PDs/APDs/CCC Chairs were asked to prioritize. Measuring the trainee’s PC competency (63.2%) was one of the areas that most needed improvement through the development of a new tool, revision of the existing instruments, or creation of alternative assessment plans. The top six common high-demand needs among all programs are listed in Table 3.

**Table 3:** Assessment needs to be expressed by PDs/APDs across 19 programs. PC: patient care, MK: medical knowledge, PBLI: practice-based learning and improvement, SBP: systems-based practice.

Priority order	Areas of improvement or needs	Competency areas	Reported percent of needs for competency measurement n: 19 n (%)
1	Alignment	PC	12 (63.2)
2	Evaluation management (Time)	PC	10 (52.6)
3	Discrete activity assessment	PC	9 (47.4)
3	Conference assessment	MK	9 (47.4)
4	Conference assessment	SBP	8 (42.1)
4	Conference assessment	PBLI	8 (42.1)
5	Discrete activity assessment	SBP	7 (36.8)
6	Alignment	PBLI	6 (31.6)

Along with measurement alignment improvements for the specific tools, the following needs were noted by the participating programs in the areas of curriculum development, competency assessment, and assessment management.

Curriculum development: Three programs reported a need to develop a more formalized curriculum both in PC and SBP competencies (15.8%). One program wanted to introduce a formal didactic curriculum for PROF 1 competency (5.3%). Another program wanted to look at the curriculum alignment regarding the MK competency, although the PDs reported insufficient resources to determine what needs to be covered in this training. Two programs expressed views on the technical and content training in both the PC and SBP competency areas.

Competency assessment: Several programs expressed a need for an expert evaluation for morbidity and mortality conferences, clinical assessment tools, a quality improvement mentor evaluation, a patient feedback form, a more formalized journal club evaluation to measure MK competency, and a lecture assessment instrument to measure the PBLI 4 competency using the Novice to Expert scale with simplified narratives of the Milestones. One program identified a need to integrate procedural competency questions into their rotation and/or program evaluation surveys. One subspecialty program reported that no sufficient national assessment guidelines existed which made the measurement of PBLI more challenging.

Assessment management: The majority of the programs expressed the need for a more functional, systematic plan for managing all the assessment and evaluation tasks to improve response rates. Some of the participants expressed their desire for a non-static evaluation format on iPads, such as a short direct observation form or a practical evaluation app for on-demand evaluation for diagnostic discussion or real-time assessment tasks.

Program Directors’ Challenges

PDs reported a variety of evaluation challenges that fell into five domains (Table 4). The most common challenges were related to difficulties in logistics and tracking evaluations of the resident management system (9 programs: 47.3%), in time management (8 programs: 42.1%), in determining and interpreting the Milestones numbers and levels (6 programs: 31.5%), in interpreting the verbiage with multi-descriptors/more than one concept (4 programs: 21%), and in matching the milestone descriptors with the labels for sub-competencies (4 programs: 21%).

**Table 4:** Program directors’ challenges in 19 programs. CCC: Clinical Competency Committee.

Domains	No. of programs n: 19 n (%)
Survey management issues (frequency, duration)	
Logistics/tracking difficulties of evaluation in the resident management system (survey’s technology platform)	10 (52.6)
Time management/time distribution per evaluation	9 (47.3)
Too many evaluations producing assessment and evaluation fatigue	2 (10.5)
Engagement	
Identifying fellows’ strengths and weaknesses, including fellow specific data	3 (15.7)
Inadequate participation in the evaluation	2 (10.5)
Difficulty in obtaining written feedback from faculty	2 (10.5)
Incomplete understanding of the content of a meaningful assessment	2 (10.5)
Training and support	
Difficulty in determining and interpreting the Milestones numbers and levels	6 (31.5)
Difficult in interpreting the verbiage with multi-descriptors	4 (21)
Variation of faculty training on assessment and feedback	2 (10.5)
Measurement errors in scoring and mismatch with the narratives	2 (10.5)
Conflicting faculty interpretation of the anchors in the evaluation	2 (10.5)
Faculty difficulty in assessing beyond direct supervision experiences	1 (5.2)
Clarity/alignment	
Matching the milestone descriptors with the labels for sub-competencies	4 (21)
Unclear descriptions that are not applicable to fellows	2 (10.5)
Trying to combine concepts in evaluations that do not fit together	2 (10.5)
Evaluating procedural skills that are not reflected in the Milestones	1 (5.2)
Difficulty mapping sub-competencies with program’s assessments	1 (5.2)
CCC function	
Faculty coming to CCC meeting unprepared	1 (5.2)
Faculty understanding the roles and functions of a CCC	1 (5.2)

## Discussion

Our study indicated that performance assessment and evaluation are very complex constructs and require multiple levels of assessment, commitment, and efforts to support valid interpretations of Milestones. Based on the viewpoints and experiences of PDs, APDs, and CCC Chairs in 19 GME programs, we found that multiple assessment methods were used to evaluate all essential competencies for measuring trainees’ performance abilities. Program needs and evaluation challenges varied, while there were some commonalities within different sub-specialties in using Milestones assessment. Furthermore, our study revealed that several factors are significantly associated with effective assessment and evaluation practices. The following considerations and insights can be critical to address or improve our current system.

Types of assessment and evaluation

The assessment and evaluation practices in our graduate programs seem to be meaningful because of the various types of direct and indirect measurement methodologies. Both direct and indirect assessments from a variety of sources with multiple assessment methods provide converging evidence of trainees’ competencies. However, when we look at the frequency from within the type of assessment practices for six core competencies, indirect assessment methods are being heavily used. Faculty evaluators need to be aware that all assessment methods have their limitations and contain some bias. Not applying a robust indirect assessment approach may result in intentional or unintentional errors from the evaluators’ side.

Traditionally, most faculty evaluators are familiar with direct assessment and evaluation methods, such as written exams (SITE, RITE), direct observations (Mini-CHECK, Simulation Labs, SCORE) demonstrations, and reports. These techniques provide a sampling of what trainees know and can do and provide strong evidence of their learning. However, using the Milestones assessment as a summative indirect evaluation, usually at the end of a rotation or training, cannot measure learning or skills in a direct way. Generally, indirect measures are not as robust as direct measures due to evaluators’ assumptions, perceptions, and opinions that may not match the reality of the actual achievement of a trainee. Although programs are using multiple tools and methods, we do not know how those are strategically measuring critical skills and behaviors of the trainees.

Program needs

Before conducting this study, we were supporting our graduate programs based on their needs and/or on-demand requests such as new assessment tool developments, revising current tools, and/or revising the goals and objectives of their curriculum. However, this study increased our awareness further in respect of each program's needs and priorities as addressed below.

Alignment

The results showed that the primary need was to improve the alignment between Milestones descriptions and competency measurement methods. Although the Milestones approach incorporates criterion-based, developmental, and work-based assessment procedures, the competencies may not be well aligned with the goals and objectives of a specific program curriculum. The methods of assessment and the skill being assessed may also be misaligned. Therefore, faculty judgment may fail to correspond with the actual performance of a trainee or the targeted objectives of a curriculum. At this point, curriculum alignment [15] is crucial to create coherence between instructional activities, learning objectives, and assessment methodologies [16,17]. This alignment will make a curriculum more functional, transparent, and dynamic while the process would help capture flaws and deficiencies within the curriculum. Further, ensuring the consistency and alignment between a program curriculum and the Milestones assessment is vital knowledge for faculty to understand how trainees’ learning is progressing. Of course, other factors may also make the Milestone assessment difficult for faculty such as obtaining reliable data on certain milestones in unsupervised practice, lack of milestones implementation guidance, and knowledge deficiencies of faculty and trainees in the understanding of the meaning of the competencies in the context of their specialty or the right time and point for measurement of performance [5,18].

Competency Assessment Tools

We believe that well-designed assessment tools have a significant impact to accurately measure targeted competencies. To meet the program needs regarding the competency assessment tools reported in the results section, we started working on them while this study was in progress. In addition, one of the results regarding needs surprised us. Several programs needed a Journal Club (JC) evaluation to measure MK competency although a JC toolkit was developed by the first author of this paper (KOL) on behalf of the GME Department. We shared this toolkit with multiple programs during a meeting and through a wiki site. It is obvious that our dissemination method has needed further follow-up. This finding leads us to consider our dissemination strategy for a new tool and reevaluate our communication strategies with the programs. For our future endeavors, we need a better dissemination plan that includes both informational and educational components to promote the new materials and the evaluation forms.

Faculty Training

The need for ongoing professional development and faculty training is highly emphasized to overcome obstacles and barriers for successful implementation of Milestones in the literature [18,19]. The results from our study also suggest that PDs/APDs and faculty evaluators need to be trained in the science of assessment and evaluation as well as the use of survey’s technology platform which would make it easier to use with the existing Milestones practices and be able to track the progress of their trainees. Critical knowledge and key behaviors for evaluators should be infused into the training content. These could include but not limited to: (i) developing evaluators’ judgment skills, (ii) knowing how to evaluate soft skills or hard skills, (iii) assessing the difficulty level of a task to be measured, (iv) translating knowledge into practice, (v) applying robust direct and indirect assessment approaches to various training environments, (vi) identifying measurement biases and error sources, (vii) standardizing how we evaluate trainees in various conditions, and (viii) analyzing responses and converting them into long-term benefits.

Medical faculties are usually in charge of planning educational experiences without specific training in medical education, and often with limited knowledge of technology and resources. Moreover, they often have had minimal experience with educational pedagogy. To increase success in assessment practices, faculty should be trained in the skills needed to design, develop, and implement a formal curriculum, including writing goals and objectives, alignment strategies, technical knowledge, and curriculum delivery methods. This training should include curriculum alignment (as mentioned above) since this process shows the relationships of all components of a curriculum from pedagogy to assessment methods [20]. Further, how to align a specific curriculum around nationally accepted standards should be covered, even though only a few programs addressed the weaknesses of the national standards.

While all these training elements mentioned above are relevant to the role of faculty educators, they cannot be accomplished in a short term through a faculty training program unless faculty development and changes are gradually and systematically implemented to create a stronger culture of assessment. Thus far, with the guidance from GME Department, the Office of Faculty Development has offered some sessions on assessment and evaluation of trainees to address some of the issues above, but we will continue providing more educational seminars and workshops until we obtain high levels of success in faculty members' assessment and evaluation practices.

Challenges

Due to the complexity involved in implementing Milestones, medical educators and program leaders have had obstacles and assessment challenges of the Milestones to assess medical trainees’ performance in various specialties [4,5,18,21]. The challenges included the need for direct observation, time constraints, faculty development, verbatim/language clarity, lack of guidance, new assessment tools, issues with measurement scales, adapting assessment efforts, and clarification of expectations. Our study also showed similar challenges, as described below.

As reported in the results section, PDs/APDs and faculty were challenged by the assessment format of the Milestones due to many factors, such as the multi-descriptors, lexical chaining (a group of semantically related words), or textual coherence (logical links or consistency between the words, sentences, and paragraphs of the text), misalignment or difficulties mapping with the sub-competencies, unclear or generic descriptions, and lack of a procedural skills evaluation component. All these ambiguities and inconsistencies, especially in the multi-descriptors (rubric format) cause difficulty for the evaluators in determining and interpreting the Milestones numbers and levels. In general, items in an assessment or evaluation tool should measure one concept at a time, not two or three. Since Milestones are in a rubric format (holistic evaluation approach), we may be able to convert it into an analytic assessment format using “behaviorally anchored rating” scales that would provide narrative descriptors of performance at various points along the scale [22]. In this way, we can create a precise, concise, and accurate description to define each competency and behavioral attribute in measuring trainees’ performance actions at different levels in both clinical and non-clinical environments. If the scale is well-aligned with the assessment task and purpose, this would eliminate different interpretations by evaluators and reduce the problems with reliability and validity [23]. Thus, many faculty and CCC Chairs have come to a consensus on what the behavioral anchors are within their specialty.

Furthermore, to eliminate the Milestones assessment challenges faced by faculty, a well-designed faculty guide should be provided with examples of behaviors, skills, measurement tips, and specific data that can be used by faculty or will assist them in deciding the competency levels of trainees. Although GME has promoted the use of the Milestones Guidebooks for programs, residents, and fellows, as well as the CCCs Guidebook compiled by ACGME [24-26], we must ensure that those resources are efficient or effective. Also, leaders in GME have been periodically attending the programs’ CCCs meetings to provide feedback to CCC Chairs and PDs about their assessment practices of the Milestones.

Surveys management and evaluation fatigue

Survey management was mentioned as both a need and a challenge in the results section. This issue is critical for monitoring, administering, and tracking the status of the assessment and evaluation activities. In this perspective, the user’s acceptance of a survey’s technology platform is a key success factor for working in a well-organized manner. However, the PDs/APDs were not completely satisfied with the survey’s technology platform which in some cases was not being used to its full potential. Any technical platform that is perceived as not being user-friendly will decrease a user’s motivation and increase frustration. To address this issue, we will continue ongoing educational training for the Program Coordinators and PDs/APDs on maximizing the technical platform. Finally, we would like to explore how we can transition to the real-time assessment of trainees’ professional knowledge, skills, and behaviors through an app-based system, as the participants expressed interest in dynamic app-based assessment methodologies.

Our study results also directed our attention to time management issues, since PDs, APDs, and faculty evaluators are physicians with busy schedules and multiple responsibilities. We may be able to address this issue by creating a better survey calendar and revisiting the timelines for assessment and evaluation activities. An evaluation timeline and management plan based on the academic year can provide a general idea of what one cycle of an assessment and evaluation looks like in a program. At the same time, it can help us distribute balanced assessment and evaluation activities in a systematic way.

Another result concerned evaluation fatigue. Although only a small percentage of programs raised the issue of evaluation fatigue, we believe it to be a common problem among faculty members due to multiple factors, such as time constraints, perceptions of survey value, over surveying, survey length, survey topic, question clarity, and question types [27,28]. We specifically found fatigue around the number of evaluations that faculty are asked to complete (due to a large number of learners in our system) as well as the need for us to appropriately target and balance which questions we need to ask of which audiences. For instance, in areas of ICS and PROF, we have a large number of sources, our own data show, whereas we have fewer sources for MK and SBS because fewer individuals have the expertise to assess these competencies. This burden may be reduced by creating a value-based evaluation model, which involves making value judgments in assessment or evaluation tasks. We are in the process of devising a value-based approach in most surveys we are implementing. We believe that identifying the values of each type of assessment and evaluation may help us collect more reliable and valid data.

In order to effectively and efficiently implement the Milestones assessments, it is crucial that we should communicate with the PDs/APDs and CCC Chairs about their personal and professional challenges by documenting and discussing their experience and concerns with the Milestones assessment. Through this structured process, we can develop a better understanding of the support they need, including the challenges and barriers that inhibit their assessment and evaluation activities. We recognize that faculty development needs around the Milestones are significant and that it is worth an investment in time and resources to perform Milestone assessments with learners successfully. ACGME has just transitioned to a new online learning portal called “Learn at ACGME.” All customizable educational resources, on-demand online courses, videos, and collaborative opportunities on this platform can contribute to educating our faculty in the science of assessment and Milestones-related issues.

Finally, we would like to note a few limitations of this study. (i) Our study participants’ understanding competency level of milestone-based assessment and their years of experience may be diverse or vary based on their time within this position. (ii) Our data are limited to only the viewpoint of PDs/APDs and CCC Chairs. We were not able to capture the program faculty perspectives on milestones assessment, and they may have different insights than PDs/APDs and CCC Chairs.

## Conclusions

Milestones assessment and evaluation in medical education can be a challenge, but a priority for many training programs. Multiple assessment methods must be utilized to evaluate all essential competencies for accurate measurement of trainees’ performance abilities. Our study provided a broad range of viewpoints, insights, and in-depth understanding of what has been currently accomplished with the milestones assessment by the graduate programs. It was also a valuable venue for bringing all 19 programs together with GME leadership to discuss important issues in their programs and creating a common language for assessing Milestones across all specialties. The results aided in devising a plan to prioritize the program needs that would lead to actions by the Department of GME in distributing supports and services to all programs equally in the areas of technical expertise, consultation, guidance, and mentoring. Although we responded to many needs of the programs, we will continue working with each program until we obtain the desired results both in improving measurement tools and the curriculum alignment with the ACGME’s core competencies and Board Specifications for all programs. While the results from our study cannot be generalized beyond our context, our collective case study can be a model for other educational programs to identify their assessment and evaluation challenges, training needs, and support priorities from their institutions or departments. In addition, the information and experiences obtained from our unique cases can be transferable to other settings or programs.

## Appendices

**Table 5:** ACGME core competencies. Source: ACGME Program Director Guide to The Common Program Requirements-2012 [24].

Competencies	Description
PC	The ability to provide PC that is compassionate, appropriate, and effective for the treatment of health problems and the promotion of health.
MK	The demonstration of knowledge about established and evolving biomedical, clinical, epidemiological, and social-behavioral sciences, as well as the application of this knowledge to PC.
PBLI	The ability to investigate and evaluate one’s care of patients, to appraise and assimilate scientific evidence, and to continuously improve patient care based on constant self-evaluation and life-long learning.
SBP	The demonstration of an awareness of and responsiveness to the larger context and system of health care and the ability to effectively call on system resources to provide care that is of optimal value.
ICS	The demonstration of interpersonal and communication skills that result in effective information exchange and collaboration with patients, their families, and professional associates.
PROF	The demonstration of a commitment to carrying out professional responsibilities, adherence to ethical principles, and sensitivity to a diverse patient population.

Questions for the template as the focused conversation guide:

1.      What are the specific program activities regarding the Milestones assessment? /What type of educational activities do you use to teach your residents and fellows about the six core competencies? (Program Activities)

2.      How do you assess the Milestone-related six core competencies in your program? And what are your current practices or techniques and assessment tools that you have utilized so far? (Current Assessment and Evaluation Tools)

3.      Do you use self-assessment in your program? If so, how do you use or evaluate a resident’s self-assessment? (Current Assessment and Evaluation Tools)

4.      How can we support or ease your Milestones assessment in your program? /What are your urgent needs to function better in the Milestone assessment endeavors? (Program Needs/Needed Assessment and Evaluation Tools)

5.      What Milestones are most difficult for you to assess your residents/fellows in your program? And why? (Challenges)

6.      What are your plans or what process do you have for resident/fellow performance improvement in your program? And tell us your future needs. (Needs)
